# Photocatalytic Activity of TiO_2_/g-C_3_N_4_ Nanocomposites for Removal of Monochlorophenols from Water

**DOI:** 10.3390/nano12162852

**Published:** 2022-08-18

**Authors:** Thawanrat Kobkeatthawin, Suwilai Chaveanghong, Jirawat Trakulmututa, Taweechai Amornsakchai, Puangrat Kajitvichyanukul, Siwaporn Meejoo Smith

**Affiliations:** 1 Center of Sustainable Energy and Green Materials and Department of Chemistry, Faculty of Science, Mahidol University, 999 Phuttamonthon Sai 4 Road, Salaya, Nakhon Pathom 73170, Thailand; 2 Center of Excellence for Innovation in Chemistry, 272 Rama VI Road, Rajthevi, Bangkok 10400, Thailand; 3 Department of Environmental Engineering, Faculty of Engineering, Chiang Mai University, 239 Huay Kaew Road, Muang District, Chiang Mai 50200, Thailand; 4 Sustainable Engineering Research Center for Pollution and Environmental Management, Faculty of Engineering, Chiang Mai University, 239 Huay Kaew Road, Muang District, Chiang Mai 50200, Thailand

**Keywords:** monochlorophenol (MCPs), graphitic carbon nitride (g-C_3_N_4_), titanium dioxide (TiO_2_), photocatalytic activity

## Abstract

This research employed g-C_3_N_4_ nanosheets in the hydrothermal synthesis of TiO_2_/g-C_3_N_4_ hybrid photocatalysts. The TiO_2_/g-C_3_N_4_ heterojunctions, well-dispersed TiO_2_ nanoparticles on the g-C_3_N_4_ nanosheets, are effective photocatalysts for the degradation of monochlorophenols (MCPs: 2-CP, 3-CP, and 4-CP) which are prominent water contaminants. The removal efficiency of 2-CP and 4-CP reached 87% and 64%, respectively, after treatment of 25 ppm CP solutions with the photocatalyst (40TiO_2_/g-C_3_N_4_, 1 g/L) and irradiation with UV–Vis light. Treatment of CP solutions with g-C_3_N_4_ nanosheets or TiO_2_ alone in conjunction with irradiation gave removal efficiencies lower than 50%, which suggests the two act synergically to enhance the photocatalytic activity of the 40TiO_2_/g-C_3_N_4_ nanocomposite. Superoxide and hydroxyl radicals are key active species produced during CP photodegradation. In addition, the observed nitrogen and Ti^3+^ defects and oxygen vacancies in the TiO_2_/g-C_3_N_4_ nanocomposites may improve the light-harvesting ability of the composite and assist preventing rapid electron-hole recombination on the surface, enhancing the photocatalytic performance. In addition, interfacial interactions between the MCPs (low polarity) and thermally exfoliated carbon nitride in the TiO_2_/g-C_3_N_4_ nanocomposites may also enhance MCP degradation.

## 1. Introduction

In past decades, public awareness of the release of pollutants such as herbicides, pesticides, and hazardous chemicals into the environment through industrial and agricultural activities was a key driver for the implementation of new legislation and environmental standards. Monochlorophenols (MCPs), including 2-chlorophenol (2-CP), 3-chlorophenol (3-CP) and 4-chlorophenol (4-CP), are pollutants being prioritized by the United States Environmental Protection Agency [1] due to their high toxicity, carcinogenicity, environmental persistence, and low biodegradability [2,3]. MCPs have been widely used as components of pesticides, herbicides, and bactericides [4,5] used on farmlands, and are also employed in the production of dyes, pharmaceuticals, and in paper processing [6]. Since MCPs may cause DNA damage resulting in carcinogenic or mutagenic effects and histopathological changes in humans and animals [7], the effective removal of MCPs from natural waterways and soils is of vital importance. Wastewater remediation methods, including physicochemical [8,9] and biological technologies [10,11], have been applied for MCPs removal; however, these are largely ineffective for water containing high concentrations of MCPs [12,13]. On the other hand, advanced oxidation processes (AOPs) based on radical reactions, such as electrocatalysis [14], Fenton oxidation [15], and photocatalysis [16,17] showed superior performance in the removal of highly persistent MCPs. Examples include the degradation of MCPs with Co/g-C_3_N_4_ using peroxymonosulfate (PMS) as the oxidant [18], and the degradation of 2-CP using CNTs/AG/ITO electrodes [19]. Furthermore, photocatalytic wastewater treatments have shown great promise due to their high efficiency, the utility of cheap radiation sources for operation, the use of ambient temperatures, and that the organic pollutants can be completely mineralized, affording CO_2_ and H_2_O [20,21]. Regarding photoactive materials, graphitic carbon nitride (g-C_3_N_4_) has recently attracted attention due to its two-dimensional structure, low cost, stability, and visible-light driven bandgap [22,23]. However, fast recombination of photogenerated electron-hole pairs on pristine g-C_3_N_4_ and its typically low specific surface area may contribute to its low photocatalytic efficiency [24] in organic compound degradation. Notwithstanding this, g-C_3_N_4_ materials have recently found uses as support materials for immobilization of other semiconductors, resulting in synergic photocatalytic performance. These synergic effects arise through improved charge separation in the electron transfer processes and a further shifting of the light absorption boundary into the visible region. While several examples of composite materials exist for the photocatalytic degradation of MCPs (Table 1), the example based on bulk g-C_3_N_4_ and TiO_2_ [25] affords only low 2-CP removal efficiency (38%) after 1 h treatment. In this study, exfoliated g-C_3_N_4_ nanosheets were applied to produce a series of TiO_2_/g-C_3_N_4_ nanohybrid photocatalysts having different weight ratios of TiO_2_, and their photocatalytic performance for the removal of MCPs from wastewater was investigated. The resulting nanocomposites were characterized in terms of structure, chemical composition, morphology, and optical properties. Identifications of active radical species generated in photocatalytic treated aqueous MCPs were carried out. An additional discussion on the utility of TiO_2_/g-C_3_N_4_ based photocatalysts for remediation of pesticide contaminated water is given based on the results reported in this research and those of recent works [26,27,28,29].

## 2. Experimental

### 2.1. Chemicals

All chemicals used in the experiments were of AR grade and used without further purification. Urea (CH_4_N_2_O), titanium (IV) oxysulfate (TiOSO_4_), 3-chlorophenol (C_6_H_5_ClO), and 4-chlorophenol (C_6_H_5_ClO) were obtained from Kemaus, Australia. 2-Chlorophenol, ammonium oxalate ((NH_4_)_2_C_2_O_4_), nitric acid (HNO_3_), and methanol (CH_3_OH) were purchased from Merck, Germany. 5,5-Dimethyl-l-pyrroline N-oxide (C_6_H_11_NO) was purchased from Cayman, Japan. Benzoquinone and isopropyl alcohol were obtained from Sigma-Aldrich, St. Louis, MO, USA. Deionized water was used throughout this study.

### 2.2. Characterization

Powder X-ray diffraction was performed to study the crystalline structure of samples using CuK_α_ radiation (λ = 1.54 Å) over a 2θ range = 10–90° (Bruker AXS, D8 advance, Karlsruhe, Germany). X-ray photon spectroscopy (XPS) was employed to determine the surface electronic state of samples (XPS; AXIS Ultra DLD, Kyoto, Japan). Raman spectra were recorded using an excitation wavelength of 875 nm (Horiba, XploRA Plus, Lille, France). Sample morphologies were visualized using transmission electron microscopy (JEM-2100 Plus, JEOL, Chiyoda, Japan) and scanning electron microscopy (SEM; Hitachi: SU8010, Matsuda, Japan). Surface area measurements were carried out by N_2_ sorption a 77 K, using a Micromeritics ASAP2060 instrument. The specific surface area was obtained by the Brunauer–Emmett–Teller (BET) method. Band gap energies of prepared samples were obtained by UV-Vis NIR spectrophotometer (Shimadzu, UV3600 plus, Tokyo, Japan). Electron-hole recombination in samples was investigated through photoluminescence (PL) spectra, obtained at an excitation wavelength of 320 nm (Horiba, FluoroMax, Longjumeau cedex, France). Electron paramagnetic resonance (EPR) signals of free radicals were recorded at ambient temperature (Bruker; Elexsys 500, Rheinstetten, Germany). The degradation of MCPs was monitored by measuring the absorbance with a UV-Vis spectrophotometer (Perkin Elmer, Lambda 800, Waltham, MA, USA). GC/MS was used for the separation and identification of photocatalytic degradation products (Agilent GC7890B–MSD5977B, Santa Clara, CA, USA) and utilized a HP-5MS UI column measuring 30 m × 0.25 mm × 0.25 µm. Operating conditions were as follows: sample injection volume 1 µL, initial oven temperature 60 °C for 1 min, followed by a temperature gradient of 20 °C/min to 300 °C.

### 2.3. Synthesis of g-C_3_N_4_

Urea powder (125 g) was added to an alumina crucible, and the powder was heated in a muffle furnace at a heating rate of 10 °C/min to 600 °C, and the sample then held at this temperature for 4 h. After cooling to room temperature, bulk g-C_3_N_4_ (pale yellow solid) was obtained (Bulk-CN). The bulk material was converted to g-C_3_N_4_ nanosheets by thermal exfoliation in the presence of nitrate. In a typical process, 2.5 g of bulk-CN was stirred in 65% HNO_3_ solution (100 mL) for 12 h. After washing with deionized water, the obtained sample was annealed at 500 °C for 4 h, and then cooled to room temperature affording g-C_3_N_4_ nanosheets (CNNS) or the exfoliated g-C_3_N_4_ material.

### 2.4. Fabrication of TiO_2_/g-C_3_N_4_ Nanocomposites

Composites containing different quantities of TiO_2_ relative to CNNS (20–50% TiO_2_ by weight) were synthesized by a hydrothermal process, with the obtained composites labelled as 20TiO_2_/CNNS, 30TiO_2_/CNNS, 40TiO_2_/CNNS and 50TiO_2_/CNNS. In this process, an appropriate quantity of TiOSO_4_ was dispersed in deionized water (100 mL) by stirring for 15 min (magnetic stirrer), and then using an ultrasonic bath (20 min). The required quantity of g-C_3_N_4_ was then added, and ultrasonication continued for a further 30 min. After this, the suspension was transferred to a Teflon-lined autoclave which was closed and then heated at 180 °C for 4 h. After cooling to room temperature, the autoclave was opened and the precipitate was collected by centrifugation, then washed two times with distilled water. The obtained powder was dried at 65 °C for 24 h before analysis. As a control, TiO_2_ in the absence of g-C_3_N_4_ was also subjected to the same process and collected as above.

### 2.5. Photocatalytic Activity Evaluation

The photocatalytic activity of samples was evaluated by their ability to degrade MCPs (2-CP, 3-CP and 4-CP) in aqueous solution, under UV–Visible light irradiation in a home-built metal photoreactor box (dimensions: 30 × 80 × 32 cm³) equipped with a W lamp (300 watts, Osram Ultra-Vitalux, Nové Zámky-Dolná kapsa, Slovakia). The emission spectrum obtain from the utilized light source was previously reported [35]. The light bulb was set at a distance of 25 cm from each sample solution, and the temperature of the sample was maintained at 35 °C using a water bath. The light intensity of 1.37 ± 0.05 klx was measured by a luxmeter (Extech 403125, Kaohsiung, Taiwan) at the sample solution. Following a previously reported protocol [26], 10 mg of catalyst was suspended in 10 mL of a 25 ppm aqueous MCP solution. The suspension was stirred for 1 h in the dark to achieve adsorption equilibrium, before light irradiation for certain period of time (30, 60, 90, and 120 min). The solution was then collected using a syringe equipped with a micropore filter (0.45 µm) to separate the photocatalyst. The concentration of MCP in the collected solution was obtained using UV–Vis spectrophotometry, from absorbance measurements at 274 (for 2-CP), 274 (for 3-CP) and 280 nm (for 4-CP). The MCPs removal efficiency was calculated via Equation (1) [26].

% Removal efficiency = 100 × ((C_0_ − C_t_)/C_0_) (1)

where: C_0_ is the initial concentration of MCPs, C_t_ is the concentration of MCPs after t minutes.

### 2.6. Identification of Active Species during Photocatalytic Degradation

Electron paramagnetic resonance (EPR) was used to detect active species such as hydroxyl (OH) and superoxide (O_2_^−^) radicals, with the assistance of 5,5-dimethyl-l-pyrroline N-oxide (DMPO). Hydroxyl radicals and superoxide radicals were trapped in aqueous solution and methanol, respectively. Samples for EPR measurements were prepared by adding 2 mg of photocatalyst to 2 mL of 25 ppm MCPs solution to form a suspension. This was stirred in the dark for 1 h to achieve adsorption equilibrium, and then 1 mL of DMPO (50 mM) in aqueous solution was added. Stirring was continued and the mixture irradiated with a W lamp (300 watt) for 60 min. The solution (200 µL) was then filtered through a syringe filter (0.45 µM) and EPR spectra were measured for this solution. For a comparison, EPR spectra were measured for the solution from treatment treated with photocatalyst in the dark and in the absence of irradiation (denoted as 0 min). Photolysis was also studied by irradiating the solution, in the absence of photocatalyst.

## 3. Result and Discussion

### 3.1. Structural and Chemical Properties

**Figure 1:**
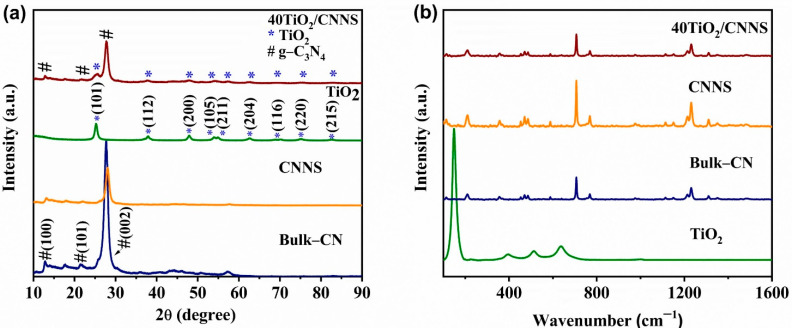
(**a**) PXRD patterns of bulk-CN, CNNS, TiO_2_, and 40TiO_2_/CNNS, (**b**) Raman spectra of bulk −CN, CNNS, TiO_2_, and 40TiO_2_/CNNS. An enlarged PXRD profile of 40TiO_2_/CNNS is provided in Figure S1.

Powder X-ray diffraction (PXRD) patterns of the bulk-CN, CNNS, TiO_2_ and 40TiO_2_/CNNS nanocomposites are shown in Figure 1a. As all nanocomposites (20TiO_2_/CNNS -50TiO_2_/CNNS) exhibit identical diffraction patterns, only that of 40TiO_2_/CNNS is shown. Bulk-CN and CNNS display three characteristic peaks at around 13.0°, 21.6°, and 27.5°, which are assigned to the (100), (101), and (002) hexagonal crystal planes (JCPDS 87-1526), respectively [36]. The sharp peak at 27.5° is attributed to interlayer stacking of aromatic rings, with the lower intensity peak at 13.0° being due to the presence of tri-s-triazine units [37,38]. The (002) peak intensity decreases significantly on conversion of bulk-CN to CNNS, and exhibits a slight shift in 2θ, reflecting the exfoliation of bulk-CN to nanosheets with shorter stacking distances between g-C_3_N_4_ layers [39,40]. Titanium dioxide exhibits diffraction peaks corresponding to the (101), (112), (200), (105), (211), (204), (116), (220), and (215) planes, consistent with the material existing as tetragonal anatase (JCPDS 021-1272) [41,42]. As expected, the patterns of g-C_3_N_4_/TiO_2_ nanocomposites exhibit peaks arising from both pure g-C_3_N_4_ and TiO_2_, and the absence of any peak shifting in the TiO_2_ peaks relative to anatase (Figure S1) indicates that coupling with g-C_3_N_4_ does not influence the TiO_2_ lattice structure, which might be beneficial regarding the photocatalytic activity of the hybrid photocatalyst.

**Figure 2:**
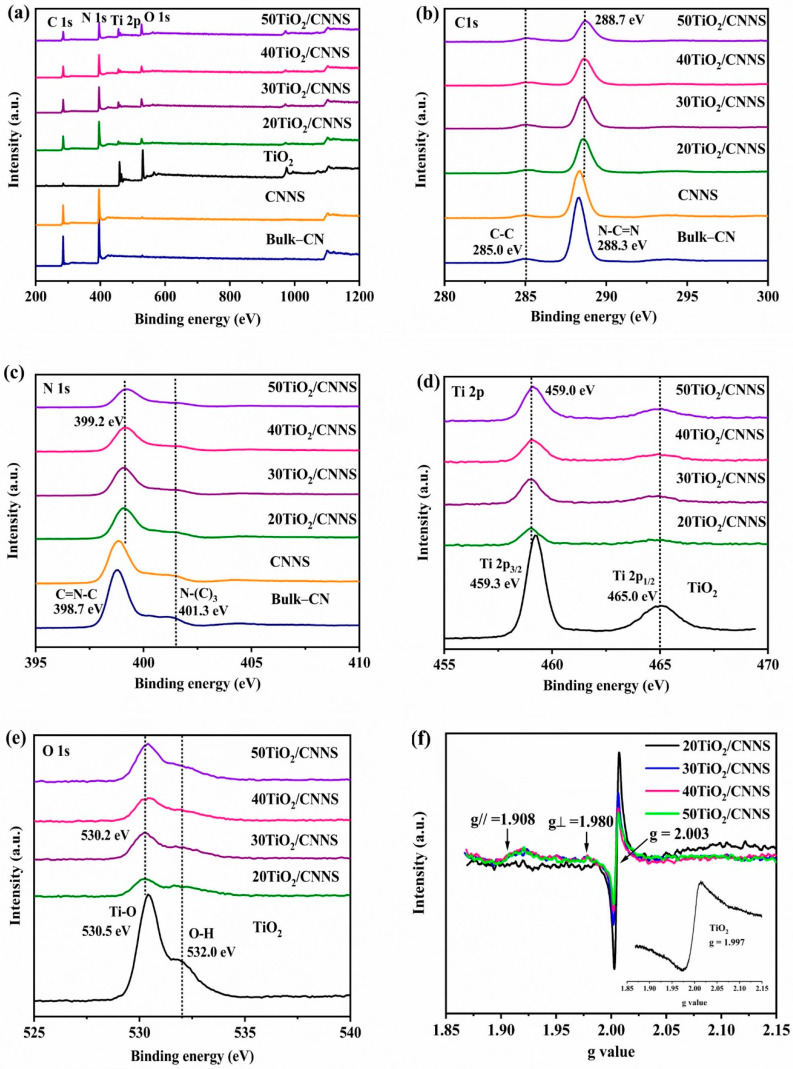
(**a**) Survey XPS spectra of precursor and nanocomposite samples, (**b**) C1s XPS spectra, (**c**) N1s XPS spectra, (**d**) Ti Figure 3b, f2p XPS spectra, (**e**) O1s XPS spectra, and (**f**) Solid phase EPR spectra of Ti^3+^-TiO_2_/CNNS nanocomposite and anatase TiO_2_.

Raman spectra of the 40TiO_2_/CNNS nanocomposite and its component materials are shown in Figure 1b. The peak at 707 cm^−1^ in CNNS and 40TiO_2_/CNNS is assigned to the signature peak of g-C_3_N_4_, which arises due to the breathing modes of tri-s-triazine structural elements. Additionally, peaks at 1230 cm^−1^ in all CN samples are consistent with the stretching vibration modes in C-N heterocycles [43,44]. Valence state and bonding information of elements in the nanocomposites and precursor materials was obtained using X-ray photoelectron spectroscopy (XPS), with survey XPS spectra shown in Figure 2a. The nanocomposites are composed of C, N, Ti, and O, confirming the existence of TiO_2_ and g-C_3_N_4_ in these materials. High resolution C1s spectra of g-C_3_N_4,_ and of the nanocomposite materials, are presented in Figure 2b. All samples exhibit two C1s peaks, ascribed to the sp^2^ C–C at 285.0 eV and sp^2^ hybridized carbon atoms (N–C=N) in aromatic rings at 288.3 eV, respectively [45,46]. In the case of N1s spectra (Figure 2c), two different peaks located around 398.7 and 401.3 eV are visible, with the main peak at 398.7 eV arising from nitrogen atoms bonded to sp^2^-hybridized carbon (C=N–C). The low intensity 401.3 eV peak is a result of N bonded to three carbon atoms N–(C)_3_ in the aromatic moieties [47].

To examine the formation of defects on exfoliation EPR spectra of bulk-CN and CNNS were recorded, and these are shown in Figure S2. The EPR signal at g = 2.003 in CNNS is of significantly higher intensity than that in bulk-CN, due to the formation of N-defects on exfoliation which increases the number of unpaired electrons on C atoms [48]. In addition, the C1s and N1s peak positions in the nanocomposites are shifted relative to those of g-C_3_N_4_, suggestive of interactions at the interface between g-C_3_N_4_ and TiO_2_ [49]. Peaks located at 459.2 and 464.9 eV in the high-resolution Ti 2p spectrum (Figure 2d) correspond to the Ti 2p_3/2_ and Ti 2p_1/2_ of TiO_2_, respectively, confirming the presence of Ti^4+^ species in TiO_2_ and its composites [50]. The O1s spectrum is given in Figure 2e, and its fitting with convolution (Figure S3) indicated three peaks with binding energies of 531.9, 530.4 and 533.23 eV which can be ascribed to oxygen bound to Ti^4+^, oxygen vacancies (V_o_) and oxygen from H_2_O, respectively [51]. The formation of Ti^3+^ in nanocomposites was confirmed by solid state EPR measurements (Figure 2f). As shown in the inset, a strong EPR signal from TiO_2_ was observed at g = 1.997, which is characteristic of Ti^3+^ defects (3d^1^, S = 1/2) [51,52]. The major signal (g = 2.003) occurs from the presence of unpaired electrons on aromatic carbon centers in g-C_3_N_4_ [53]. The EPR signals at g_//_= 1.908 and g_Ʇ_ = 1.980 were assigned to Ti^3+^ defects in TiO_2._, with the reduction in Ti^4+^ to Ti^3+^ occurring by loss of oxygen from the surface during high temperature hydrothermal treatment [54,55]. No Ti^3+^ signals were observed for the 20TiO_2_/CNNS nanocomposite which reflects its low photocatalytic MCPs degradation efficiency.

**Figure 3:**
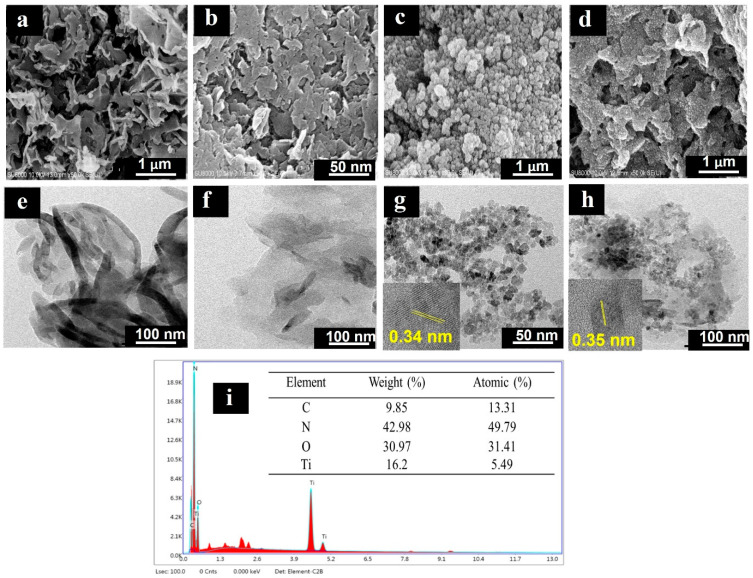
(**a**) SEM and TEM images of (**a**,**e**) bulk-CN, (**b**,**f**) CNNS, (**c**,**g**) TiO_2_, (**d**,**h**) 40TiO_2_/CNNS, and (**i**) EDX-SEM data for 40TiO_2_/CNNS.

The morphologies of bulk-CN, CNNS, TiO_2_ and 40TiO_2_/CNNS nanocomposites, as imaged using SEM and TEM, are shown in Figure 3. Bulk-CN (Figure 3a,e) presents a lamellar structure in line with previous reports [56]. Annealing of bulk-CN at 500 °C results in the formation of CNNS, which takes the form of nanosheets of smaller particles (Figure 3b,f). Results from N_2_ adsorption–desorption isotherms indicate that the specific surface area of CNNS (91.6 m^2^ g^−1^) is higher than that of Bulk-CN (68.2 m^2^ g^−1^). Graphitic carbon nitride layers exfoliate during high temperature thermal oxidation as these are held together by weak intermolecular forces (Van der Waals forces and hydrogen bonding). In addition, the presence of HNO_3_ assists layer separation due to the intercalation of nitrate ions, which causes interplanar swelling. Exfoliation results in reduced layer thickness and smaller particle size, which increases the specific surface area [57,58,59]. Pure TiO_2_ consists of agglomerated spherical-like particles around 10 nm in diameter (Figure 3c,g). The interplanar distance of 0.34–0.35 nm (inset) in these particles is in agreement with the d-spacing of the (101) planes in anatase TiO_2_ [60]. SEM and TEM images of 40TiO_2_/CNNS are suggestive of TiO_2_ particles being dispersed on the surface of g-C_3_N_4_ (Figure 3d,h) which should enhance the transfer of photogenerated electrons and result in greater photocatalytic activity. In addition, the EDX spectrum in Figure 3i highlights the elemental composition of TiO_2_/g-C_3_N_4_ nanocomposites, which confirms the formation of heterojunctions between g-C_3_N_4_ and TiO_2_.

### 3.2. Optical Properties

**Figure 4:**
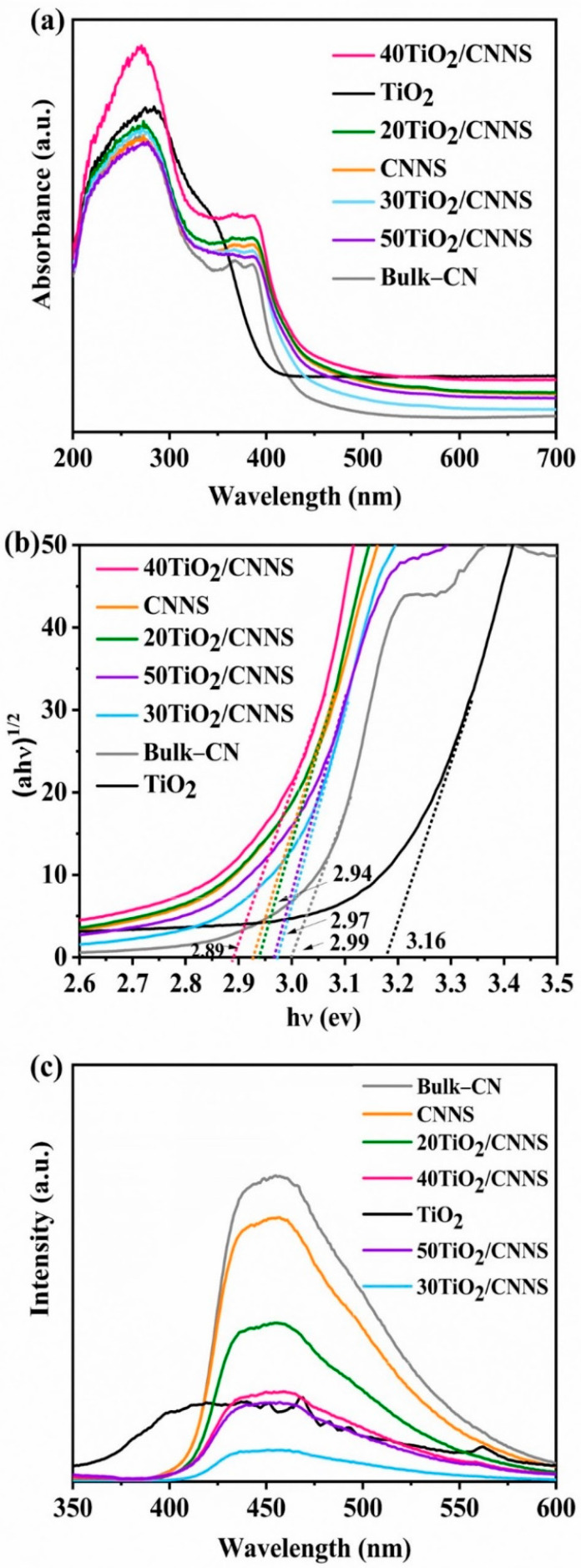
(**a**) Ultraviolet–visible (UV–Vis) diffuse reflectance spectra, (**b**) corresponding Tauc plot displaying band gaps of g-C_3_N_4_, TiO_2_ and composites, and (**c**) photoluminescence spectra of bulk-CN, CNNS, and nanocomposite materials. Extrapolated dotted lines (in Figure 4b) are added to indicate energy bandgap of each sample.

The UV–Vis DRS spectra of g-C_3_N_4_, TiO_2_ and nanocomposites are shown in Figure 4a. Pristine g-C_3_N_4_ has a strong absorption band in the UV–Vis region with an absorption edge close to 430 nm [61]. Anatase TiO_2_ exhibits similar absorption behavior, with an adsorption edge at ca. 410 nm [62]. After coupling with g-C_3_N_4_, the absorption edge of composites exhibits a red shift compared with that of TiO_2_, as the presence of Ti^3+^ and oxygen vacancies allows for a relaxation of selection rules governing transitions in TiO_2_, resulting in improved absorption profiles [63]. Band gap energies were calculated using the Tauc plot (Figure 4b) and through Equation (2).

Ahν = A(hν − E_g_)^1/2^ (2)

In this equation, α, h, ν, A, and E_g_ are the optical absorption coefficient, Planck constant, photon frequency constant, and band gap energy, respectively [64].

From Figure 4b, the band gap energy of g-C_3_N_4_ nanosheet is estimated to be 2.93 eV (slightly narrower than that of bulk g-C_3_N_4_, 2.99 eV), which is consistent with past work [65]. The band gap energy of TiO_2_ obtained (3.16 eV) differs slightly from that previously reported for anatase TiO_2_ materials (3.2 eV), possibly due to the presence of Ti^3+^ defects [66]. As shown in Figure 4a, 40TiO_2_/CNNS absorbs in the visible region and exhibits the lowest bandgap energy (2.89 eV, Figure 4b), which are both advantageous features for photocatalysts. Electron-hole recombination was studied using photoluminescence analysis, with the results shown in Figure 4c. Under excitation at 320 nm, the emission peak of g-C_3_N_4_ occurs at 457 nm, which is consistent with UV–Vis DRS results. Bulk-CN and CNNS show high PL intensities, suggesting fast recombination of electron-hole pairs. On the other hand, TiO_2_ shows a broad emission peak at 410 nm and lower intensity than that of g-C_3_N_4_ or CNNS. After hybridization of g-C_3_N_4_ and TiO_2_, all composites show much weaker emission peaks, implying that recombination of charge carriers may be effectively inhibited by Ti^3+^-V_o_.

### 3.3. Photocatalytic Degradation of MCPs

**Figure 5:**
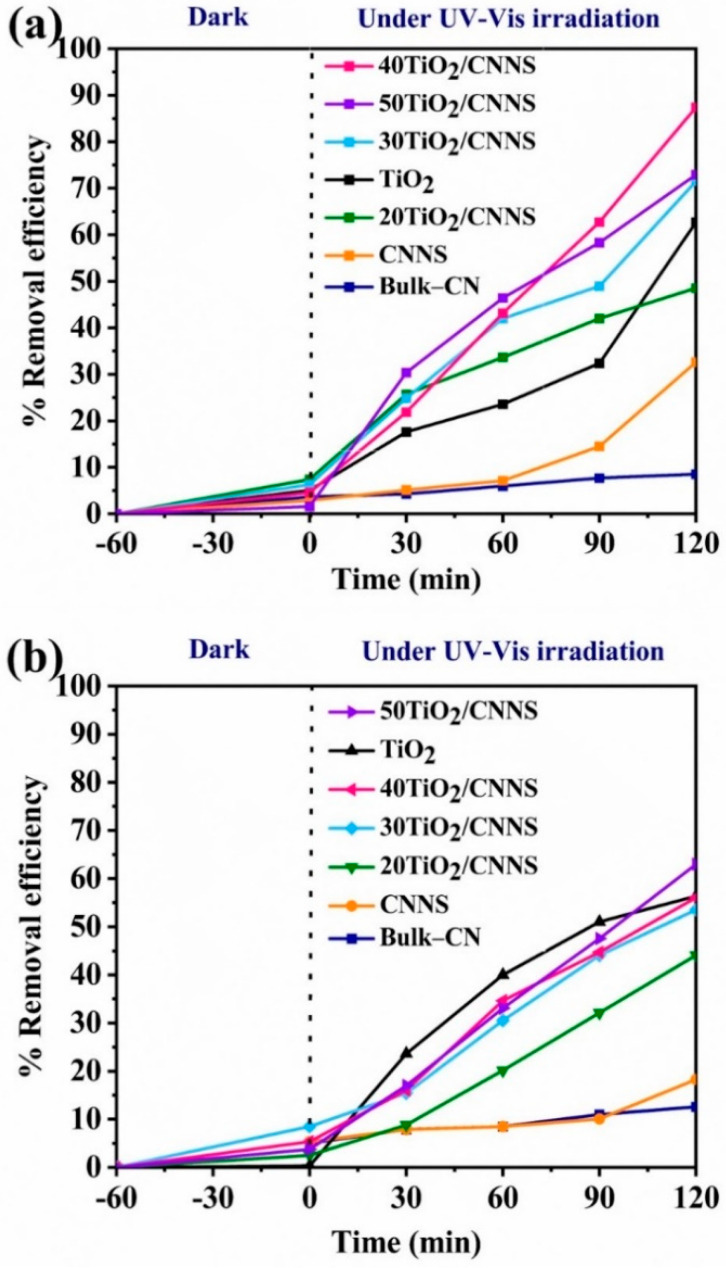

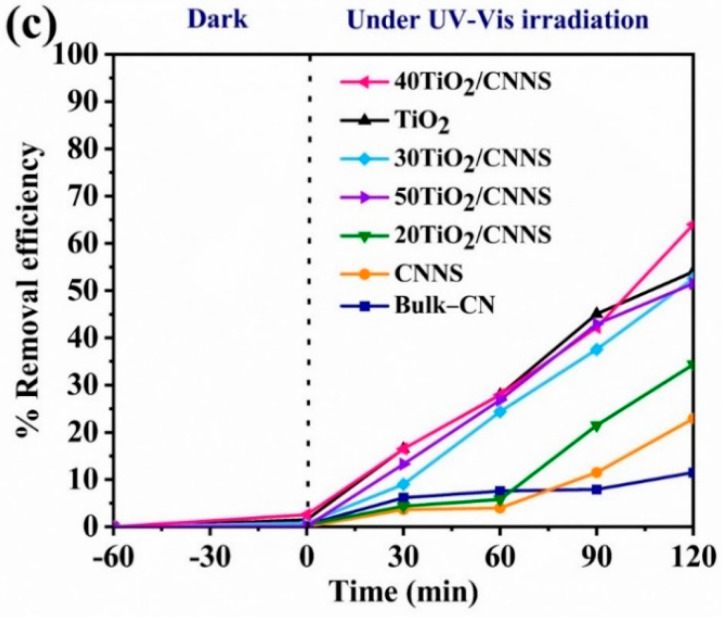
Photocatalytic degradation profiles for (**a**) 2-chlorophenol, (**b**) 3-chlorophenol, and (**c**) 4-chlorophenol when treated with g-C_3_N_4_, TiO_2_, and composites under UV–Vis irradiation (25 ppm pollutant, 1 g/L catalyst loading). Plots with error bars from triplicate measurements are given in Figure S5.

The photocatalytic performance of nanocomposites for degradation of MCPs under UV–Vis irradiation, as reported in terms of % removal efficiencies, are shown in Figure 5. Notably, as a control, photolysis was performed with 2-CP, 3-CP and 4-CP under UV–Vis irradiation without catalyst addition for 2 h (Figure S4). In these cases, absorbance values of the phototreated MCPs solutions showed no significant differences from those from untreated MCPs solutions, suggesting negligible removal of MCPs in the absence of photocatalyst. These findings agree with those from previous work [67], and reflect the structural stability of MCPs.

Results in Figure 5 show that Bulk-CN, CNNS, and 20TiO_2_/CNNS materials are only marginally effective for MCPs degradation, with removal efficiencies being less than 50% after 2 h. This is presumably due to electron-hole recombination, as implied by PL results (Figure 4b).

Notably, CNNS exhibits higher photocatalytic activity than bulk g-C_3_N_4_. Exfoliation results in nanosheets with a greater surface area and the addition of N-defects, both of which enhance photocatalytic activity [68,69]. Defect modification is one promising way to enhance the photocatalytic activity of g-C_3_N_4_, as it results in band gap narrowing and extension of the light absorption range [69]. The recombination of the photoexcited electrons and holes can also be inhibited through the midgap state generated by the introduction of the N defects, leading to enhancements in photocatalytic activity. All other composites than 20TiO_2_/CNNS and TiO_2_ alone show photocatalytic efficiencies greater than 50% after 2 h. The 40TiO_2_/CNNS nanocomposite is the most active in degradation of 2-CP and 4-CP, giving removal efficiencies of 87% and 64% after 2 h, respectively. These results illustrate the synergistic effect of TiO_2_ and g-C_3_N_4_, and the importance of Ti^3+^-Vo defects, to the photocatalytic performance. As indicated earlier, 40TiO_2_/CNNS has the lowest band gap energy (2.89 eV), allowing the harvesting of light in the visible region, and the introduction of Ti^3+^ provides hole traps to promote charge separation and suppress recombination. From UV–Vis DRS results (Figure 5), 30TiO_2_/CNNS and 50TiO_2_/CNNS show wider band gaps compared with 20Ti/CNNS and CNNS.

While the 50TiO_2_/CNNS displays strong light absorption in the visible region and contain Ti^3+^ defects, no such defects were observed in 20TiO_2_/CNNS which underlines the importance of both band gap and defects on photocatalytic activity. Results from UV–Vis DRS spectra indicate that 50TiO_2_/CNNS exhibits a wider band gap than 40TiO_2_/CNNS. Possible reasons for 40TiO_2_/CNNS exhibiting the highest performance could be its low band gap energy (2.89 eV) allowing harvesting of light in the visible region, and the presence of Ti^3+^ species, providing hole traps to promote charge separation and suppress charge recombination. The nitrogen defects in CNNS and the interactions between the CNNS and TiO_2_ particles (discussed herein) may account for the superior performance of these composites relative to bulk g-C_3_N_4_/TiO_2_ in the photocatalytic degradation of 2-CP [25]. Notably, the photocatalytic activities for 2-CP degradation are higher than that obtained for 3-CP and 4-CP [70], potentially due to stronger adsorption of 2-CP over the other isomers. The photocatalytic removal efficiency of 2-, 3-, and 4-CP over the 40TiO_2_/CNNS nanocomposite was ca. 87%, 64%, and 22%, respectively (Figure 5), which agrees with previous findings [70] such that the removal efficiencies of 2-CP > 3-CP > 4-CP. Additionally, the position of the Cl substituent on the aromatic ring can affect the photocatalytic degradation profile of MCPs, as ortho and para C-Cl bonds can be cleaved more easily than those of meta-Cl substituents as a result of inductive and mesomeric effects [16].

**Figure 6:**
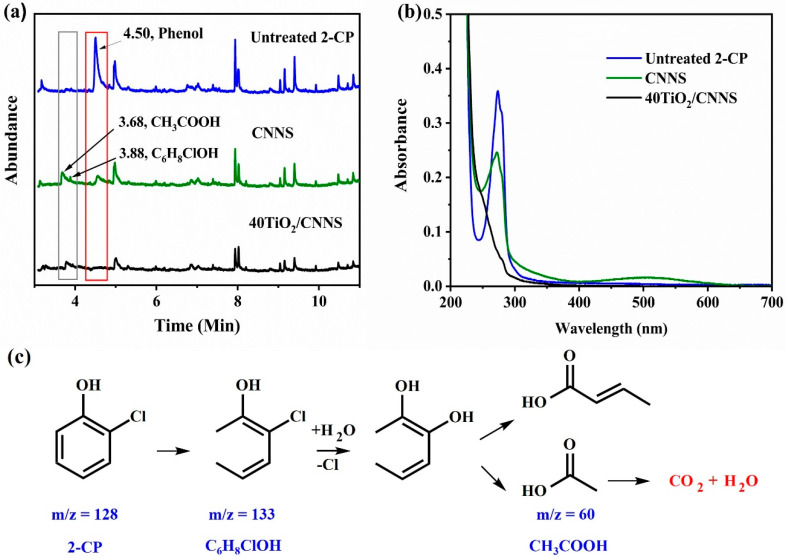
(**a**) GC traces of untreated 2-CP, 2-CP when treated with CNNS, and 2-CP when treated with 40TiO_2_/CNNS. (**b**) UV–Vis spectra of 2-CP, and 2-CP treated with CNNS and 40TiO_2_/CNNS. (**c**) A possible degradation pathway (from MS data) for 2-CP in the presence of 40TiO_2_/CNNS.

To analyze the degradation pathway of 2-chlorophenol, GC-MS was employed to visualize and identify potential breakdown products. As shown in Figure 6a, the GC trace of untreated 2-CP shows a peak corresponding to the phenol at a retention time of 4.50 min. The intensity of this peak decreases on treatment with CNNS and is completely absent after treatment with 40TiO_2_/CNNS, which is in agreement with UV–Vis spectra obtained for solutions treated with this photocatalyst (Figure 6b). Intermediate products were observed at retention times of 3.68 (*m*/*z* = 63) and 3.88 (*m*/*z* = 133) min after treatment with CNNS or 40TiO_2_/CNNS which, based on fragmentation data, could be acetic acid (CH_3_COOH) [70,71] and C_6_H_8_ClOH, respectively. A possible degradation pathway for 2-CP, under optimum conditions in the presence of 40TiO_2_/CNNS, is proposed in Figure 6c.

**Figure 7:**
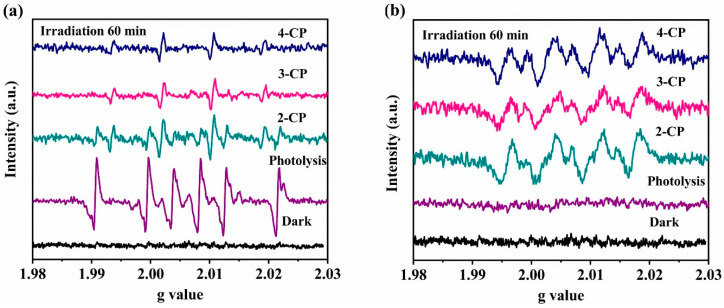
DMPO spin trapping EPR spectra for investigating the generation of (**a**) hydroxyl radicals in aqueous CP solutions treated with 40TiO_2_/CNNS and (**b**) superoxide radicals in methanolic CP solutions treated with 40TiO_2_/CNNS.

Moreover, the generation of radical species in the CP solutions treated with photocatalyst was probed using EPR spectroscopy with DMPO as the radical spin trapping agent (Figure 7). The presence of hydroxyl radicals was tested under aqueous conditions whereas superoxide radicals were trapped in methanol solution. Under photolytic conditions, both radicals were observable (Figure 7). However, in the dark, or under photocatalytic conditions using 40TiO_2_/CNNS, neither radical species could be detected. By contrast, signals characteristic of the formation of DMPO-OH and DMPO-O_2_^−^ adducts were observed after light irradiation for 60 min, which confirms the presence of OH and O_2_^−^ in the photocatalytic mechanism.

### 3.4. Photocatalytic Mechanism

To further describe the photocatalytic mechanism for the degradation of 2-CP using 40TiO_2_/CNNS, the CB and VB edge potentials of g-C_3_N_4_ and TiO_2_ were calculated, at the point of zero charge, using Equations (3) and (4) [72].

E_CB_ = X − E_c_ − 1/2E_g_ (3)

E_VB_ = E_CB_ + E_g_ (4)

In Equations (3) and (4), X is the absolute electronegativity of the atom semiconductor, being 5.8 eV for TiO_2_ and 4.73 eV for g-C_3_N_4_. E_c_ represents the energy of free electrons on the hydrogen scale (4.5 eV) [73] and E_g_ is the band gap energy of the semiconductor. From the UV–Vis DRS spectra (Figure 4b), the E_g_ of g-C_3_N_4_ is 2.94 eV and TiO_2_ is 3.16 eV. Therefore, the reductive potentials of the conduction band (CB) are −0.28 and −1.24 V for TiO_2_ and g-C_3_N_4_, and the oxidation potentials of the valence band (VB) of TiO_2_ and g-C_3_N_4_ are +2.88 and +1.70 V, respectively. Under light exposure, electrons are excited from the VB to the CB in TiO_2_ (Ti^3+^and V_o_). Photogenerated holes thus remain in the TiO_2_ valence band, while photogenerated electrons transfer from the CB of Ti^3+^ and O_v_ to the VB of g-C_3_N_4_. Electrons in the VB of g-C_3_N_4_ are further excited into the CB, which leads to enhanced separation between the photo-induced charge carrier and an increase in the redox ability. The electrons stored in the CB of g-C_3_N_4_ are then trapped on the surface and reduced to superoxide radical ions (O_2_^−^), while the holes in the VB of TiO_2_ can oxidize OH^−^ or H_2_O to form hydroxyl radicals (OH), which further react with chlorophenol resulting in CO_2_ and H_2_O products [72,73]. Therefore, the formation of Ti^3+^ and V_o_ is a major factor dictating the photocatalytic activity of TiO_2_/CNNS nanocomposites. Classified as a type II heterojunction, the electrons on CB of g-C_3_N_4_ are transferred to TiO_2_ which undergo a reduction reaction, whereas holes on the surface of TiO_2_ are transferred to the g-C_3_N_4_ VB for oxidation reaction. This mechanism suggested the lowering of reduction ability of photogenerated electrons and the weakened oxidation capability of holes. The holes in the VB of g-C_3_N_4_ cannot adsorb water molecules near the surface of g-C_3_N_4_ to generate hydroxyl radicals (OH) because the reduction potential of g-C_3_N_4_ (+1.70 V) is less than that required to oxidize H_2_O to OH (+1.99 V). However, OH species were detected by EPR from the 2-CP solution on treatment with 40TiO_2_/g-C_3_N_4_. Therefore, the Z-scheme mechanism of 40Ti/CNNS is proposed (Figure S6), quite similar to a previous report [26].

Comparatively, the content of thermally exfoliated g-C_3_N_4_ required in the TiO_2_/CNNS nanocomposites produced in this work (weight ratio of TiO_2_:CNNS = 40:60) is much higher than photocatalysts affording high removal efficiencies (>80%) of imidacloprid from aqueous solution (TiO_2_:CNNS; weight ratio of TiO_2_:CNNS = 96:4, [26]). Thus, the optimum CNNS content in the TiO_2_/CNNS nanocomposites is varied for effective photodegradation of each pesticide. The relatively high CNNS content may enhance the number of π-π interactions at the interface between the aromatic pollutants (MCPs) and the exfoliated carbon nitride promoting surface reactions, resulting in a more facile degradation of MCPs over the TiO_2_/CNNS composites described here. The polarity of MCPs (log *K*_ow_ ≈ 2.5 [74]) is quite low compared with imidachloprid (log *K*_ow_ = 0.57 [75]), and the adsorption of MCPs is preferable on the CNNS surface (rather than TiO_2_), promoting the surface photoreactions. Recent works utilizing g-C_3_N_4_ based photocatalysts for the removal of pesticides (endosulfan [27], and 2,4-dinitrophenylhydrazine or 2,4-DNP [28]) from water suggest that further modifications of the oxide semiconductor (e.g., adding Cu or N dopants) could also further synergically enhance the performance of the photocatalysts. The high g-C_3_N_4_ content (30% by weight) in the TiO_2_/g-C_3_N_4_ based composites also correlates well with the low polarity of 2,4-DNPH [28]. Therefore, the synergetic effects obtained by incorporation of g-C_3_N_4_ with an oxide semiconducting material could be the results of the improved stability of photogenerated electrons and holes, as well as the increased adsorption preference sites of molecular pollutants of low polarity on the composite surface.

## 4. Conclusions

Exfoliated g-C_3_N_4_ nanosheets (CNNS) were produced via HNO_3_ assisted thermal exfoliation of urea-derived bulk g-C_3_N_4_. The nanosheets showed larger specific surface areas than that of the bulk g-C_3_N_4_ and contains N vacancy defects, promoting enhanced photocatalytic activity. The addition of TiO_2_ (20–50% by weight) followed by hydrothermal processing results in TiO_2_/g-C_3_N_4_ nanocomposites, having improved photocatalytic performance over bulk g-C_3_N_4_, exfoliated g-C_3_N_4_, or TiO_2_ alone for the degradation of MCPs. The highest removal efficiency of 87% was achieved from the treatment of 2-chlorophenol with 40TiO_2_/CNNS, followed by UV irradiation for 2 h. Such photocatalytic performance can be attributed to the existence of nitrogen, Ti^3+^, and V_o_ defects in the nanocomposites, enhancing the separation efficiency of photogenerated carriers and charge recombination suppression. It was found that OH and O_2_^−^ radicals are active species in the photocatalytic degradation of 2-CP. These thermally exfoliated carbon nitride materials may provide preferable adsorption sites for MCPs, enhancing the photocatalytic performance of TiO_2_/g-C_3_N_4_ nanocomposites. Further investigation of the nanocomposites obtained by greener routes or non-toxic precursors should be carried out to optimize the sustainability of photocatalytic processes.

## Figures and Tables

**Table 1:** Comparison of photocatalytic activities of semiconductors based on g-C_3_N_4_ utilized in the degradation of aqueous MCPs.

Pollutant	Catalyst	Source	Dosage	MCP Treatment Time (h)	% Removal Efficiency	Ref
2-CP	Bulk g-C_3_N_4_ TiO_2_ TiO_2_/bulk g-C_3_N_4_	Xe (150 W)	4 g/L	1	9% 3% 38%	[25]
	g-C_3_N_4_ OH-C_3_N_4_	W (300 W)	0.5 g/L, 10 ppm	5	95% 85%	[29]
	10% g-C_3_N_4_/FST 15% g-C_3_N_4_/FST g-C_3_N_4_ 5% g-C_3_N_4_/FST FST (FST: fibrous silica titania)	W (400 W)	0.37 g/L, 10 ppm	4	93% 70% 67% 49% 40%	[30]
3-CP	TiO_2_/N-TiO_2_ TiO_2_	Fluorescent lamp (154 W)	0.2 g/L, 10 ppm	5	77% 36%	[31]
	TiO_2_/N-TiO_2_ TiO_2_	Incandescent lamp (100 W)		24	30% 12%	
4-CP	g-C_3_N_4_/Bi_5_Nb_3_O_15_ g-C_3_N_4_	-	1 g/L, 10 ppm	1	100% 72%	[32]
	g-C_3_N_4_ g-C_3_N_4_/ZnWO_4_(1:1) g-C_3_N_4_/ZnWO_4_(2:1) g-C_3_N_4_/ZnWO_4_(3:1)	Xe (500 W)	0.2 g/L, 10 ppm	1.67	45% 62% 78% 88%	[33]
	C/ZnO/g-C_3_N_4_	Solar (300 W) UV (400 W)	0.2 g//L, 10 ppm	5	92% 72%	[34]

## Data Availability

Data are contained within the article or supplementary material.

## Supplementary Materials

The following supporting information can be downloaded at: https://www.mdpi.com/article/10.3390/nano12162852/s1, Figure S1: Enlarged powder X-ray diffraction profile of the 40TiO_2_/CNNS composite; Figure S2: Solid ESR spectra of bulk-CN (red) and CNNS (black); Figure S3: Convolution fitting of XPS spectrum of O1s in TiO_2_; Figure S4: UV–Vis spectra of photolysis study in the absence of catalyst of (a) 2-CP, (b) 3-CP and (c) 4-CP aqueous solutions. Figure S5. Time-dependent photocatalytic degradation of a) 2-chlorophenol, b) 3-chlorophenol, and c) 4-chlorophenol when treated with g-C3N4, TiO_2_ and composites under UV-Vis irradiation (25 ppm pollutant, 1g/L catalyst loading). Figure S6. Photocatalytic mechanism of utilizing 40TiO_2_/CNNS for MCPs degradation

## References

[1] Jin M., Chen X., Pan B. (2006). Simultaneous determination of 19 chlorophenols in water by liquid chromatography-mass spectrometry with solid-phase extraction. J. Liq. Chromatogr. Relat. Technol..

[2] Jiang C., Yu H., Lu Y., Zhu S., Geng Z., Huo M., Wang X. (2018). Preparation of spike-like palladium nanoparticle electrode and its dechlorination properties. Thin Solid Films.

[3] Lan S., Feng J., Xiong Y., Tian S., Liu S., Kong L. (2017). Performance and mechanism of piezo-catalytic degradation of 4-Chlorophenol: Finding of effective piezo-dechlorination. Environ. Sci. Technol..

[4] Yang C.-H., Lee C.-M. (2008). Pentachlorophenol contaminated groundwater bioremediation using immobilized *Sphingomonas* cells inoculation in the bioreactor system. J. Hazard. Mater..

[5] Onkani S.P., Diagboya P.N., Mtunzi F.M., Klink M.J., Olu-Owolabi B.I., Pakade V. (2020). Comparative study of the photocatalytic degradation of 2-chlorophenol under UV irradiation using pristine and Ag-doped species of TiO_2_, ZnO and ZnS photocatalysts. J. Environ. Manag..

[6] Diagboya P.N., Olu-Owolabi B.I., Adebowale K.O. (2016). Distribution and interactions of pentachlorophenol in soils: The roles of soil iron oxides and organic matter. J. Contam. Hydrol..

[7] Iqbinosa E.O., Odjadjare E.E., Chigor V.N., Igbinosa I.H., Emoghene A.O., Ekhaise F.O., Igiehon N.O., Idemudia O.G. (2013). Toxicological Profile of Chlorophenols and Their Derivatives in the Environment: The Public Health Perspective. Sci. World J..

[8] Kus’mierek K. (2016). The removal of chlorophenols from aqueous solutions using activated carbon adsorption integrated with H_2_O_2_ oxidation. React. Kinet. Mech. Catal..

[9] Soto M.L., Moure A., Domínguez H., Parajó J.C. (2011). Recovery, concentration and purification of phenolic compounds by adsorption: A review. J. Food Eng..

[10] Lin H.-Y. (2017). Adsorption and biodegradation of 2-chlorophenol by mixed culture using activated carbon as a supporting medium-reactor performance and model verification. Appl. Water Sci..

[11] Olaniran A.O., Igbinosa E.O. (2011). Chlorophenols and other related derivatives of environmental concern: Properties, distribution and microbial degradation processes. Chemosphere.

[12] González L.F., Sarria V., Sánchez O.F. (2010). Degradation of chlorophenols by sequential biological-advanced oxidative process using Trametes pubescens and TiO_2_/UV. Biores. Technol..

[13] Pedroza A.M., Mosqueda R., Alonso-Vante N., Rodríguez-Vázquez R. (2007). Sequential treatment via Trametes versicolor and UV/TiO_2_/RuxSey to reduce contaminants in waste water resulting from the bleaching process during paper production. Chemosphere.

[14] Shu X., Yang O., Yao F., Zhong Y., Ren W., Chen F., Sun J., Ma Y., Fu Z., Wang D. (2019). Electrocatalytic hydrodechlorination of 4-chlorophenol on Pd supported multi-walled carbon nanotubes particle electrodes. Chem. Eng. J..

[15] Kavitha V., Palanivelu K. (2003). Degradation of 2-Chlorophenol by Fenton and Photo-Fenton Processes-A Comparative Study. J. Environ. Sci. Health A.

[16] Aroh A.O., Gimba C.E., Omoniyi K.I., Abba H., Yilleng M.T. (2019). Comparison of photocatalytic degradation of 4-chlorophenol and 3-chlorophenol using silver/palladium nanoparticles doped on TiO_2_. IJARBAS.

[17] Sharma A., Lee B.-K. (2016). Rapid photo-degradation of 2-chlorophenol under visible light irradiation using cobalt oxide-loaded TiO_2_ /reduced graphene oxide nanocomposite from aqueous media. J. Environ. Manag..

[18] Xie M., Tang J., Kong L., Lu W., Natarajan V., Zhu F., Zhan J. (2019). Cobalt doped g-C_3_N_4_ activation of peroxymonosulfate for monochlorophenols degradation. Chem. Eng. J..

[19] Liu H., Zhang Z., Ren M., Guan J., Lu N., Qu J., Yuan X., Zhang Y.-N. (2018). Preparation of the CNTs/AG/ITO electrode with high electro-catalytic activity for 2-chlorophenol degradation and the potential risks from intermediates. J. Hazard. Mater..

[20] Ahmad R., Ahmad Z., Khan A.U., Mastoi N.R., Aslam M., Kim J. (2016). Photocatalytic systems as an advanced environmental remediation: Recent developments, limitations and new avenues for applications. J. Environ. Chem. Eng..

[21] Kumar A., Pandey G. (2017). A review on the factors affecting the photocatalytic degradation of hazardous materials. Mater. Sci. Eng. C.

[22] Gao M., Feng J., Zhang Z., Gu M., Wang J., Zeng W., Lv Y., Ren Y., Wei T., Fan Z. (2018). Wrinkled ultrathin graphitic C_3_N_4_ nanosheets for photocatalytic degradation of organic wastewater. ACS Appl. Nano Mater..

[23] Hong Y., Liu E., Shi J., Lin X., Sheng L., Zhang M., Wang L., Chen J. (2019). A direct one-step synthesis of ultrathin g-C_3_N_4_ nanosheets from thiourea for boosting solar photocatalytic H_2_ evolution. Int. J. Hydrogen Energy.

[24] Fe J., Yu J., Jiang C., Cheng B. (2018). g-C_3_N_4_-based heterostructured photocatalysts. Adv. Energy Mater..

[25] Zada A., Ali N., Subhan F., Anwar N., Shah M.I.A., Ateeq M., Hussain Z., Zaman K., Khan M. (2019). Suitable energy platform significantly improves charge separation of g-C_3_N_4_ for CO_2_ reduction and pollutant oxidation under visible-light. Prog. Nat. Sci..

[26] Kobkeatthawin T., Trakulmututa J., Amornsakchai T., Kajitvichyanukul P., Smith S.M. (2022). Identification of active species in photodegradation of aqueous imidacloprid over g-C_3_N_4_/TiO_2_ Nanocomposites. Catalysts.

[27] Nekooie R., Ghasemi J.B., Badiei A., Shamspur T., Mostafavi A., Moradian S. (2022). Design and synthesis of g-C_3_N_4_/(Cu/TiO_2_) nanocomposite for the visible light photocatalytic degradation of endosulfan in aqueous solutions. J. Mol. Struct..

[28] Dong S., Chen S., He F., Li J., Li H., Xu K. (2022). Construction of a novel N-doped oxygen vacancy-rich TiO_2_ N-TiO_2−X_/g-C_3_N_4_ S-scheme heterostructure for visible light driven photocatalytic degradation of 2,4-dinitrophenylhydrazine. J.Alloys Compd..

[29] Zizhen L., Meng X., Zhang Z. (2019). Fabrication of surface hydroxyl modified g-C_3_N_4_ with enhanced photocatalytic oxidation activity. Catal. Sci. Technol..

[30] Azami M.S., Jalil A.A., Hitam C.N.C., Hassan N.S., Mamat C.R., Adnan R.H., Chanlek N. (2020). Tuning of the electronic band structure of fibrous silica titania with g-C_3_N_4_ for efficient Z-scheme photocatalytic activity. Appl. Surf. Sci..

[31] Mozia S., Bubacz K., Janus M., Morawski A.W. (2012). Decomposition of 3-chlorophenol on nitrogen modified TiO_2_ photocatalysts. J. Hazard. Mater..

[32] Zhang S., Yang Y., Guo Y., Guo W., Wang M., Guo Y., Huo M. (2013). Preparation and enhanced visible-light photocatalytic activity of graphitic carbon nitride/bismuth niobite heterojunctions. J. Hazard. Mater..

[33] Rathi V., Panneerselvam A., Sathiyapriya R. (2020). Graphitic carbon nitride (g-C_3_N_4_) decorated ZnWO_4_ heterojunctions architecture synthesis, characterization and photocatalytic activity evaluation. Diam. Relat. Mater..

[34] De Sousa J.G.M., da Silva T.V.C., de Moraes N.P., da Silva M.L.C.P., da Silva Rocha R., Landers R., Rodrigues L.A. (2020). Visible light-driven ZnO/g-C_3_N_4_/carbon xerogel ternary photocatalyst with enhanced activity for 4-chlorophenol degradation. Mater. Chem. Phys..

[35] Pennetta A., Di Masi S., Piras F., Lü X., Li J., De Benedetto G.E., Mele G. (2020). TiO_2_@lipophilic porphyrin composites: New insights into tuning the photoreduction of Cr(VI) to Cr(III) in aqueous phase. J. Compos. Sci..

[36] Zou L.-R., Huang G.-F., Li D.-F., Liu J.-H., Pan A.-L., Huang W.-Q. (2016). A facile and rapid route for synthesis of g-C_3_N_4_ nanosheets with high adsorption capacity and photocatalytic activity. RSC Adv..

[37] Yang Y., Chen J., Mao Z., An N., Wang D., Fahlman B.D. (2017). Ultrathin g-C_3_N_4_ nanosheets with an extended visible light-responsive range for significant enhancement of photocatalysis. RSC Adv..

[38] Fina F., Callear S.K., Carins G.M., Irvine J.T.S. (2015). Structural investigation of graphitic carbon nitride via XRD and neutron diffraction. Chem. Mater..

[39] Ma Y., Liu E., Hu X., Tang C., Wan J., Li J., Fan J. (2015). A simple process to prepare few-layer g-C_3_N_4_ nanosheets with enhanced photocatalytic activities. Appl. Surf. Sci..

[40] Xu J., Zhang L., Shi R., Zhu Y. (2013). Chemical exfoliation of graphitic carbon nitride for efficient heterogeneous photocatalysis. J. Mater. Chem. A..

[41] Li W., Liang L., Hu A., Huanga Z., Zhou Y.N. (2014). Generation of oxygen vacancies in visible light activated one-dimensional iodine TiO_2_ photocatalysts. RSC Adv..

[42] Majumdar S., Mahanta D. (2020). Deposition of an ultra-thin polyaniline coating on a TiO_2_ surface by vapor phase polymerization for electrochemical glucose sensing and photocatalytic degradation. RSC Adv..

[43] Wang M., Ma F., Wang Z., Hu D., Xu X., Hao X. (2018). Graphitic carbon nitride, a saturable absorber material for the visible waveband. Photonics Res..

[44] Fan C., Miao J., Xu G., Liu J., Lv J., Wu Y. (2017). Graphitic carbon nitride nanosheets obtained by liquid stripping as efficient photocatalysts under visible light. RSC Adv..

[45] Shen L., Xing Z., Zou J., Li Z., Wu X., Zhang Y., Zhu Q., Yang S., Zhou W. (2017). Black TiO_2_ nanobelts/g-C_3_N_4_ nanosheets Laminated heterojunctions with efficient visible-light-driven photocatalytic performance. Sci. Rep..

[46] Zhang Q., Ma L., Shao M., Huang J., Ding M., Deng X., Wei X., Xu X. (2014). Anodic Oxidation Synthesis of One-Dimensional TiO_2_ Nanostructures for Photocatalytic and Field Emission Properties. J. Nanomater..

[47] Ren B., Wang T., Qu G., Deng F., Liang D., Yang W., Liu M. (2018). In situ synthesis of g-C_3_N_4_/TiO_2_ heterojunction nanocomposites as a highly active photocatalyst for the degradation of Orange II under visible light irradiation. Environ. Sci. Pollut. Res..

[48] Wang J., Gao B., Dou M., Huang X., Ma Z. (2020). A porous g-C_3_N_4_ nanosheets containing nitrogen defects for enhanced photocatalytic removal meropenem: Mechanism, degradation pathway and DFT calculation. Environ. Res..

[49] Jiang D., Sun X., Zhang H., Wang K., Shi L., Du F. (2020). Nanotube confinement-induced g-C_3_N_4_/TiO_2_ nanorods with rich oxygen vacancies for enhanced photocatalytic water decontamination. Appl. Phys. A.

[50] Du X., Bai X., Xu L., Yang L., Jin P. (2020). Visible-light activation of persulfate by TiO_2_/g-C_3_N_4_ photocatalyst toward efficient degradation of micropollutants. Chem. Eng. J..

[51] Cheng D., Li Y., Yang L., Luo S., Yang L., Luo X., Luo Y., Li T., Gao J., Dionysiou D.D. (2019). One step reductive synthesis of Ti^3+^ self–doped elongated anatase TiO_2_ nanowires combined with reduced graphene oxide for adsorbing and degrading waste engine oil. J. Hazard. Mater..

[52] Swaminathan J., Ravichandran S. (2018). Insights into the defect-centered electrocatalytic behavior of reduced titania (TiO_1.23_). J. Phys. Chem. C.

[53] Kong L., Zhang X., Wang C., Xu J., Du X., Li L. (2018). Ti^3+^ defect mediated g-C_3_N_4_/TiO_2_ Z-scheme system for enhanced photocatalytic redox performance. Appl. Surf. Sci..

[54] Mohajernia S., Andryskova P., Zoppellaro G., Hejazi S., Kment S., Zboril R., Schmidt J., Schmuki P. (2020). Influence of Ti^3+^ defect-type on heterogeneous photocatalytic H_2_ evolution activity of TiO_2_. J. Mater. Chem. A.

[55] Xiong L.-B., Li J.-L., Yang B., Yu Y. (2012). Ti^3+^ in the surface of Titanium Dioxide: Generation, properties and photocatalytic application. J. Nanomater..

[56] Kathiresan V., Rajarathinam T., Lee S., Kim S., Lee J., Thirumalai D., Chang S.-C. (2020). Cost-effective electrochemical activation of graphitic carbon nitride on the glassy carbon electrode surface for selective determination of serotonin. Sensors.

[57] Che H., Liu L., Che G., Dong H., Liu C., Li C. (2019). Control of energy band, layer structure and vacancy defect of graphitic carbon nitride by intercalated hydrogen bond effect of NO_3_^−^toward improving photocatalytic performance. Chem. Eng. J..

[58] Sun H., Zhou X., Zhang H., Tu W. (2017). An efficient exfoliation method to obtain graphitic carbon nitride nanosheets with superior visible-light photocatalytic activity. Int. J. Hydrogen Energy.

[59] Dong F., Li Y.H., Wang Z.Y. (2015). Enhanced visible light photocatalytic activity and oxidation ability of porous graphene-like g-C_3_N_4_ nanosheets via thermal exfoliation. Appl. Surf. Sci..

[60] Maurya N.I.C., Singh S., Gupta A.K., Srivastava P., Bahadur L. (2017). N/Al-incorporated TiO_2_ nanocomposites for improved device performance of a dye-sensitized solar cell. Energy Environ. Sci..

[61] Yun Y.-J., He J.-Y., Zhang D., Wang X.-J., Zhao J., Liu R.-H., Li F.-T. (2020). Simultaneous construction of dual-site phosphorus modified g-C_3_N_4_ and its synergistic mechanism for enhanced visible-light photocatalytic hydrogen evolution. Appl. Surf. Sci..

[62] Zhang H., Liu F., Wu H., Cao X., Sun J., Lei W. (2017). In situ synthesis of g-C_3_N_4_/TiO_2_ heterostructures with enhanced photocatalytic hydrogen evolution under visible light. RSC Adv..

[63] Zhang X., Cai M., Cui N., Chen G., Zou G., Zhou L. (2020). One-Step Synthesis of b-N-TiO_2_/C Nanocomposites with high visible light photocatalytic activity to degrade microcystis aeruginosa. Catalysts.

[64] Dong C., Ma Z., Qie R., Guo X., Li C., Wang R., Shi Y., Dai B., Jia X. (2017). Morphology and Defects Regulation of Carbon Nitride by Hydrochloric Acid to Boost Visible Light Absorption and Photocatalytic Activity. Appl. Catal. B.

[65] Ye C., Li J.-X., Li Z.-J., Li X.-B., Fan X.-B., Zhang L.-P., Chen B., Tung C.-H., Wu L.-Z. (2015). Enhanced driving force and charge separation efficiency of protonated g-C_3_N_4_ for photocatalytic O_2_ evolution. ACS Catal..

[66] Ren R., Wen Z., Cui Z., Hou Y., Guo X., Chen J. (2015). Controllable synthesis and tunable photocatalytic properties of Ti^3+^-doped TiO_2_. Sci. Rep..

[67] Zhu M., Lu J., Dong L., Hu S., Peng S., Zhu C. (2022). Photochemical transformations of 2, 6-dichlorophenol and 2-chlorophenol with superoxide ions in the atmospheric aqueous phase. J. Mol. Struct..

[68] Liu X., Zhang Q., Cui Z., Ma F., Guo Y., Wang Z., Liu Y., Zheng Z., Cheng H., Dai Y. (2022). Morphology and defects design in g-C_3_N_4_ for efficient and simultaneous visible-light photocatalytic hydrogen production and selective oxidation of benzyl alcohol. Int. J. Hydrogen Energy..

[69] Cai H., Han D., Wang X., Cheng X., Liu J., Jia L., Ding Y., Liu S., Fan X. (2020). High specific surface area defective g-C_3_N_4_ nanosheets with enhanced photocatalytic activity prepared by using glyoxylic acid mediated melamine. Mater. Chem. Phys..

[70] Sun N., Qu Y., Yang C., Yang Z., Yan R., Zhang W.E.Z., Li Z., Li H., Khan I., Sun R. (2020). Efficiently photocatalytic degradation of monochlorophenol on in-situ fabricated BiPO_4_/β-Bi_2_O_3_ heterojunction microspheres and O_2_^−^free hole induced selective dechloridation conversion with H_2_ evolution. Appl. Catal. B.

[71] Ba-Abbad M.M., Takriff M.S., Kahum A.A.H., Mohamad A.B., Benamor A., Mohammad A.W. (2017). Solar photocatalytic degradation of 2-chlorophenol with ZnO nanoparticles: Optimisation with D-optimal design and study of intermediate mechanisms. Environ. Sci. Pollut. Res..

[72] Bi X., Yu S., Liu E., Liu L., Zhang K., Zang J., Zhao Y. (2020). Construction of g-C_3_N_4_/TiO_2_ nanotube arrays Z-scheme heterojunction to improve visible light catalytic activity. Colloids Surf. A.

[73] Liao W., Murugananthan M., Zhang Y. (2015). Synthesis of Z-scheme g-C_3_N_4_–Ti^3+^/TiO_2_ material: An efficient visible light photoelectrocatalyst for degradation of phenol. Phys. Chem. Chem. Phys..

[74] Núñez-Gaytán A.N., Vera-Ávila L.E., Covarrubias-Herrera M.d.R. (2008). On-line methodology for the trace level determination of the chlorinated phenol family in water samples. J. Mex. Chem. Soc..

[75] Moza P.N., Hustert K., Feicht E., Kettrup A. (1998). Photolysis of imidacloprid in aqueous solution. Chemosphere.

