# Supplementary material for: Photocatalytic Activity of TiO2/g-C3N4 Nanocomposites for Removal of Monochlorophenols from Water
Source: Nanomaterials (Basel). 2022 Aug 18;12(16):2852. doi: 10.3390/nano12162852 (PMC9414261; doi:10.3390/nano12162852)
Supplement: Supplementary file 1 [file nanomaterials-12-02852-s001.zip › nanomaterials-1838989-supplementary.pdf]

Article

Photocatalytic Activity of TiO_2/g-C_3N_4 Nanocomposites for Removal of Monochlorophenols from Water

Thawanrat Kobkeatthawin [1], Suwilai Chaveanghong [1], Jirawat Trakulmututa [1], Taweechai Amornsakchai [1,2], Puangrat Kajitvichyanukul [3,4,*] and Siwaporn Meejoo Smith [1,*]

[1] Center of Sustainable Energy and Green Materials and Department of Chemistry, Faculty of Science, Mahidol University, 999 Phuttamonthon Sai 4 Road, Salaya, Nakhon Pathom 73170, Thailand
[2] Center of Excellence for Innovation in Chemistry, 272 Rama VI Road, Rajthevi, Bangkok 10400, Thailand
[3] Department of Environmental Engineering, Faculty of Engineering, Chiang Mai University, 239 Huay Kaew Road, Muang District, Chiang Mai 5020, Thailand
[4] Sustainable Engineering Research Center for Pollution and Environmental Management, Faculty of Engineering, Chiang Mai University, 239 Huay Kaew Road, Muang District, Chiang Mai 50200, Thailand
* Correspondence: puangrat.k@cmu.ac.th (P.K.); siwaporn.smi@mahidol.edu (S.M.S.); Tel.: +66-61-6598715 (P.K); +66-93-5939449 (S.M.S.)

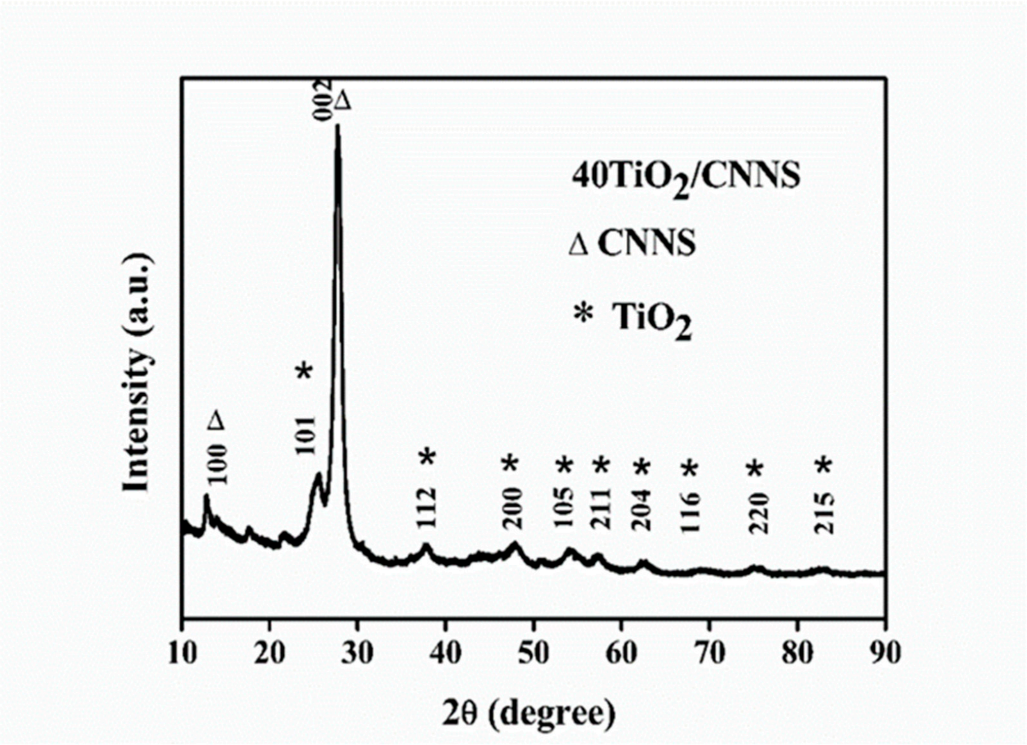

Figure S1. Enlarged powder X-ray diffraction profile of the $40TiO_2$/CNNS composite.

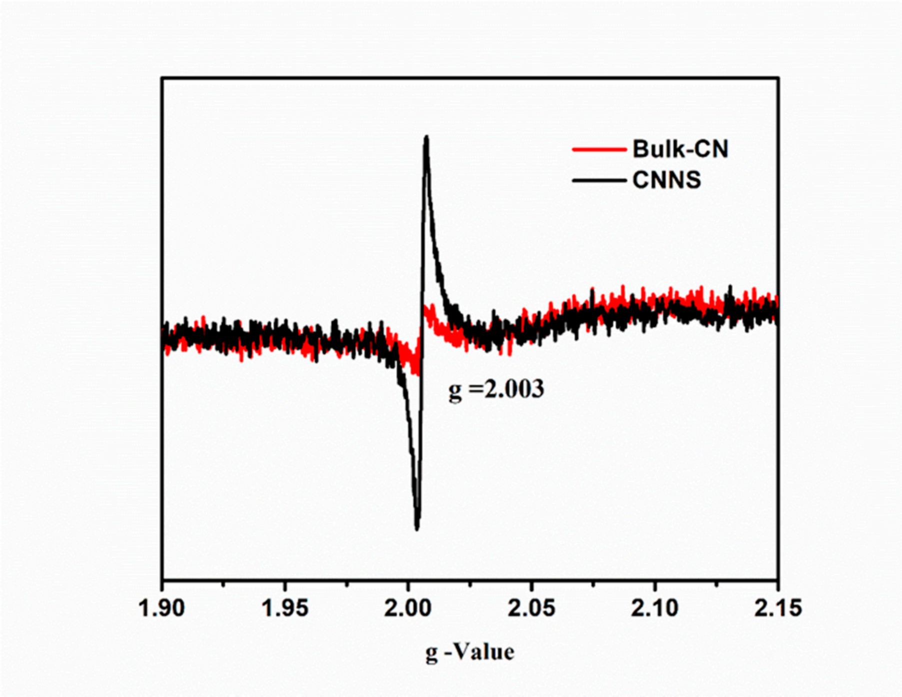

Figure S2. Solid phase ESR spectra of bulk-CN (red) and CNNS (black).

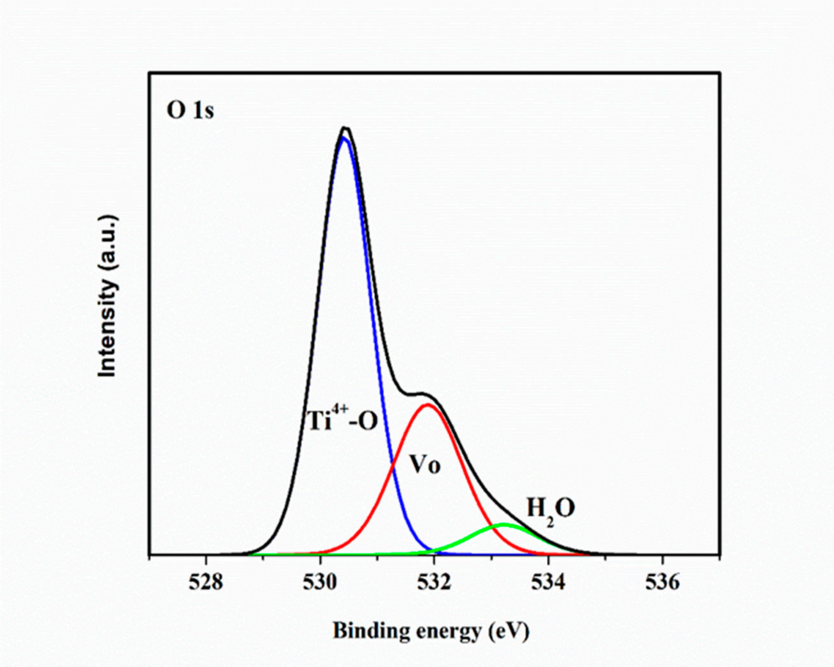

Figure S3. Convolution fitting for the O 1s XPS spectrum in TiO2.

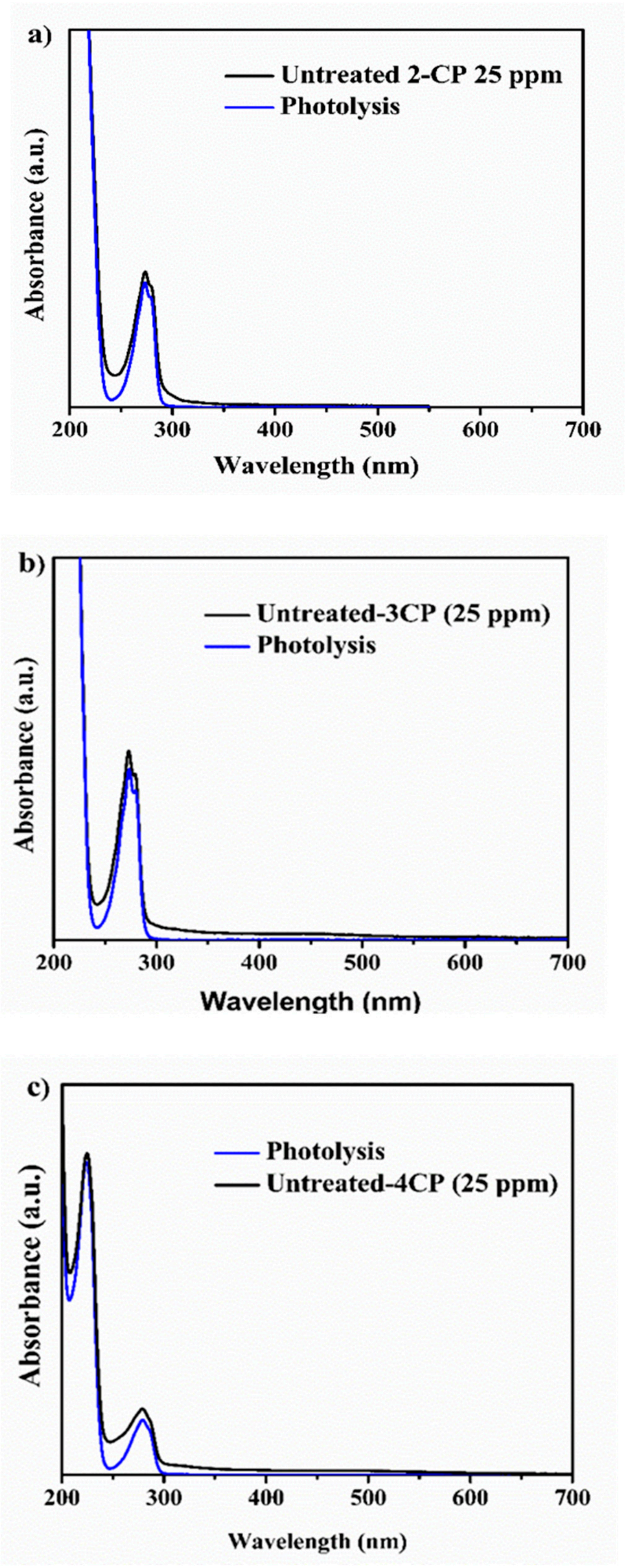

Figure S4. UV-vis spectra for photolysis of (**a**) 2-CP, (**b**) 3-CP and (**c**) 4-CP aqueous solutions in the absence of catalyst.

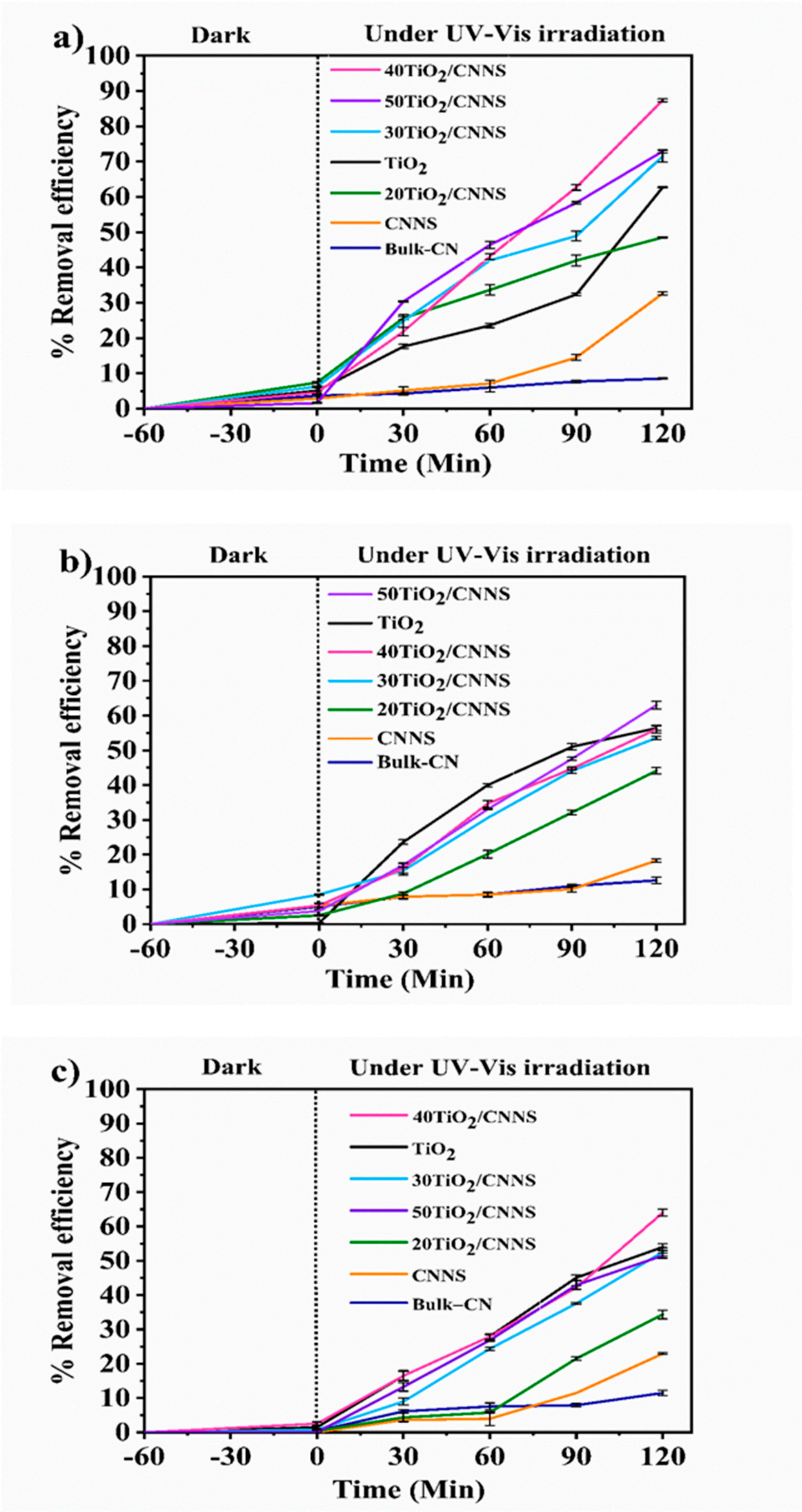

Figure S5. Time-dependent photocatalytic degradation of (**a**) 2-chlorophenol, (**b**) 3-chlorophenol, and (**c**) 4-chlorophenol when treated with g-C3N4, TiO2 and composites under UV-Vis irradiation (25 ppm pollutant, 1g/L catalyst loading).

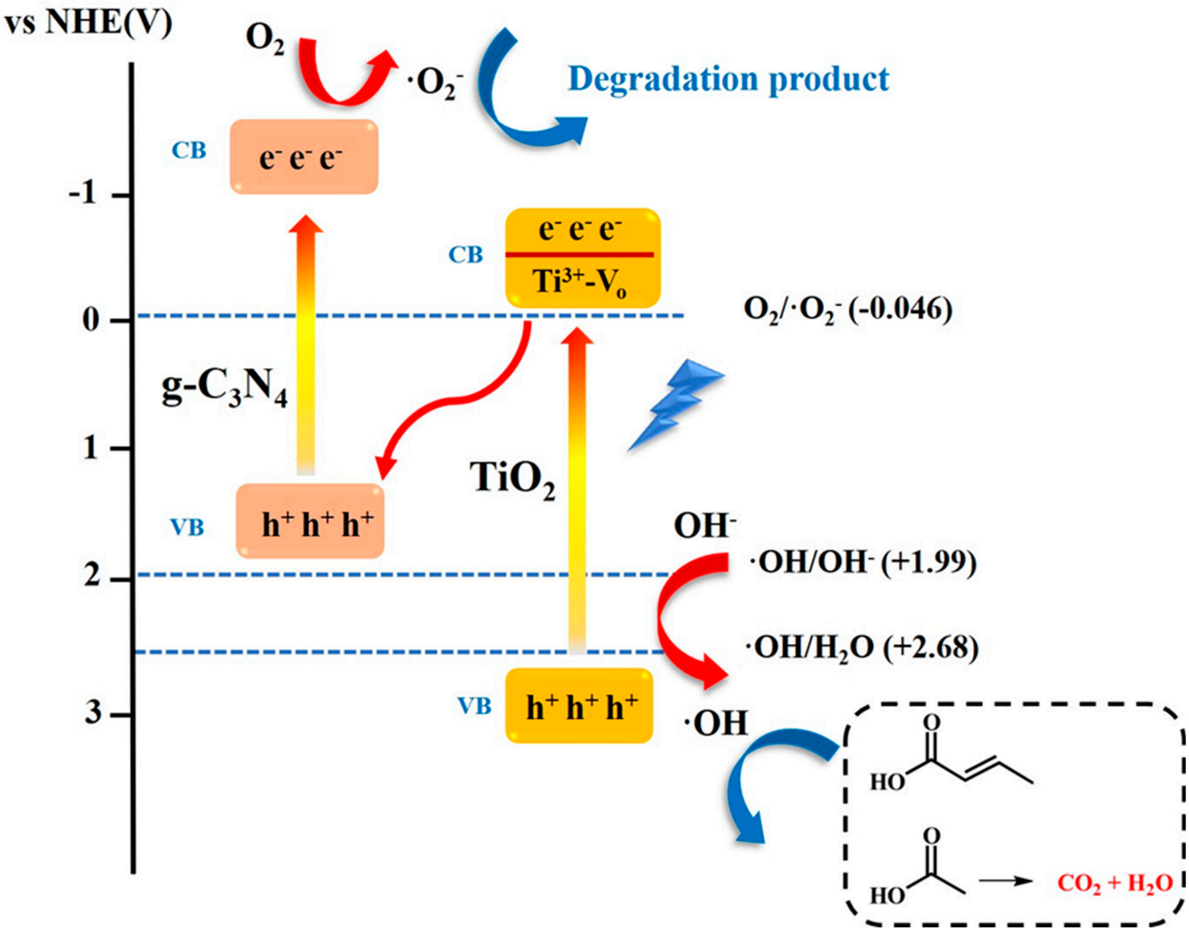

Figure S6. Photocatalytic mechanism of utilizing 40TiO2/CNNS for MCPs degradation.
